# Ambient temperature enhanced freezing tolerance of *Chrysanthemum dichrum CdICE1* Arabidopsis via miR398

**DOI:** 10.1186/1741-7007-11-121

**Published:** 2013-12-19

**Authors:** Yu Chen, Jiafu Jiang, Aiping Song, Sumei Chen, Hong Shan, Huolin Luo, Chunsun Gu, Jing Sun, Lu Zhu, Weimin Fang, Fadi Chen

**Affiliations:** 1 College of Horticulture, Nanjing Agricultural University, Nanjing, Jiangsu 210095, China; 2 Grass Research Centre, Institute of Botany, Jiangsu Province & Chinese Academy of Sciences, Nanjing 210014, Jiangsu China

**Keywords:** *Chrysanthemum dichrum*, CdICE1, Freezing tolerance, miR398, CSD

## Abstract

**Background:**

ICE (Inducer of *CBF* Expression) family genes play an important role in the regulation of cold tolerance pathways. In an earlier study, we isolated the gene *CdICE1* from *Chrysanthemum dichrum* and demonstrated that freezing tolerance was enhanced by *CdICE1* overexpression. Therefore, we sought to determine the mechanism by which *ICE1* family genes participate in freezing tolerance.

**Results:**

Using EMSA (Electrophoretic Mobility Shift Assay) and yeast one-hybrid assays, we confirmed that CdICE1 binds specifically to the MYC element in the *CdDREBa* promoter and activates transcription. In addition, overexpression of *CdICE1* enhanced *Arabidopsis* freezing tolerance after transition from 23°C to 4°C or 16°C. We found that after acclimation to 4°C, CdICE1, like *Arabidopsis* AtICE1, promoted expression of *CBF*s (*CRT/DRE Binding Factor*) and their genes downstream involved in freezing tolerance, including *COR15a* (*Cold-Regulated 15a*), *COR6.6*, and *RD29a* (*Responsive to Dessication 29a*). Interestingly, we observed that *CdICE1*-overexpressing plants experienced significant reduction in miR398. In addition, its target genes *CSD1* (*Copper/zinc Superoxide Dismutase 1*) and *CSD2* showed inducible expression under acclimation at 16°C, indicating that the miR398-CSD pathway was involved in the induction of freezing tolerance.

**Conclusions:**

Our data indicate that *CdICE1*-mediated freezing tolerance occurs via different pathways, involving either CBF or miR398, under acclimation at two different temperatures.

## Background

Low temperatures adversely affect plant quality and productivity and function as a determinant of geographical distribution and growth [1-3]. Plants achieve cold tolerance following gradual exposure to low but non-freezing temperatures, a phenomenon called cold acclimation [4-6]. Cold acclimation is accompanied by changes at the physiological, molecular and biochemical levels [7,8].

Low temperatures initiate signaling pathways that control the expression of genes encoding determinants necessary for chilling tolerance [4]. Until now, the ICE1-CBF (Inducer of *CBF* Expression - CRT/DRE Binding Factor)-cold-response pathway has been one of the dominant cold signaling mechanisms mediating cold tolerance in *Arabidopsis*[1,2,9,10]. Cold-regulated genes (*COR*) encode functional hydrophilic proteins, controlling cell osmoregulation and stabilization under freezing stress [11,12]. DRE/CRT *cis*-elements containing the core sequence CCGAC have been identified from these *COR* promoters [13,14]. Transcription factors known as CBFs (CRT binding factors) or DREB1s (DRE binding factors) induce transcription of downstream COR genes via interaction with DRE/CRT elements [15-17]. The genes encoding CBF transcription factors are up-regulated by cold. The three CBF genes encoding DREB1B/CBF1, DREB1A/CBF3, and DREB1C/CBF2 in *Arabidopsis* play a role in the cold acclimation pathway [16]. Numerous reports have demonstrated that *CBF* overexpression alleviated damage associated with freezing stress in *Arabidopsis*, rice and non-model plants [15,18,19].

Several factors involved in regulation of *CBF/DREB1* expression have been identified genetically in *Arabidopsis*. Direct regulators of *CBF/DREB1* expression include *HOS1* (high expression of osmotically responsive genes) [9], *ICE1* (inducer of CBF/DREB1 expression 1) [1] and *MYB15*[20]. The *ICE1* gene encodes a MYC-like bHLH transcription factor that binds directly to canonical MYC *cis*-elements (CANNTG) in the *CBF3/DREB1A* promoter [1]. ICE2 encodes a homolog of ICE1, and primarily influences the expression of *CBF1/DREB1B* but not that of *CBF3/DREB1A*[21]. Overexpression of two ICEs has been associated with enhanced chilling tolerance in *Arabidopsis*, rice, apples and tobacco [1,2,22,23]. Interestingly, ICE1/SCREAM is also involved in stomatal differentiation, suggesting that ICE1 mediates transcriptional regulation of environmental adaptation and stomatal development in plants [24]. In addition, protein interaction analysis reveals that ICE1 post-translational modification occurs during cold acclimation. Freezing tolerance is negatively regulated by HOS1-induced degradation of ICE1 and positively regulated by SIZ1-mediated sumoylation and stabilization of ICE1 [9,10]. Recent data indicate that serine 403 of ICE1 plays a role in the regulation of transactivation and cold-induced degradation via the ubiquitin/26S proteasome pathway, which is probably mediated by HOS1 [25]. Further investigation revealed that several jasmonate ZIM-domain (JAZ) proteins, the repressors of jasmonate signaling, physically interact with ICE1 and ICE2 transcription factors, decreasing the freezing stress response of *Arabidopsis*[26].

MicroRNAs (miRNAs), a class of small non-protein coding RNAs containing 20 to 24 nucleotides (nt), have been increasingly investigated as key regulators of gene expression [27,28]. Recent evidence indicates that plant miRNAs play a role in biotic and abiotic stress responses [29-31]. Cold-responsive miRNAs in different species enable development of breeding strategies for cold tolerance [30,32-34]. The miR398 is a repressor of Cu-Zn superoxide dismutase genes (At1g08830, *CSD1*; At2g28190, *CSD2*), which act as reactive oxygen species (ROS) scavengers [35]. MiR398 is regulated in response to oxidative stress, salt, abscisic acid (ABA), sucrose treatment and different ambient temperatures, resulting in an immediate change in *CSD* levels [35-40]. Over expression of *CSD* protects plants from oxidative stress and enhances freezing stress tolerance in transgenic plants [35]. A recent report suggested that miR398-CSD positively regulated heat tolerance [41].

The exact miR398-CSD pathway involved in the mechanism of freezing tolerance, however, is not completely understood.

In a previous investigation of chrysanthemum freezing tolerance, we isolated *CdICE1* from *Chrysanthemum dichrum*[42]. In our present study, we further explored *CdICE1* functions under two different cold acclimation conditions, which revealed that CdICE1 mediates freezing tolerance via CBF and miR398 pathways.

## Results

### *CdICE1* expression

**Figure 1:**
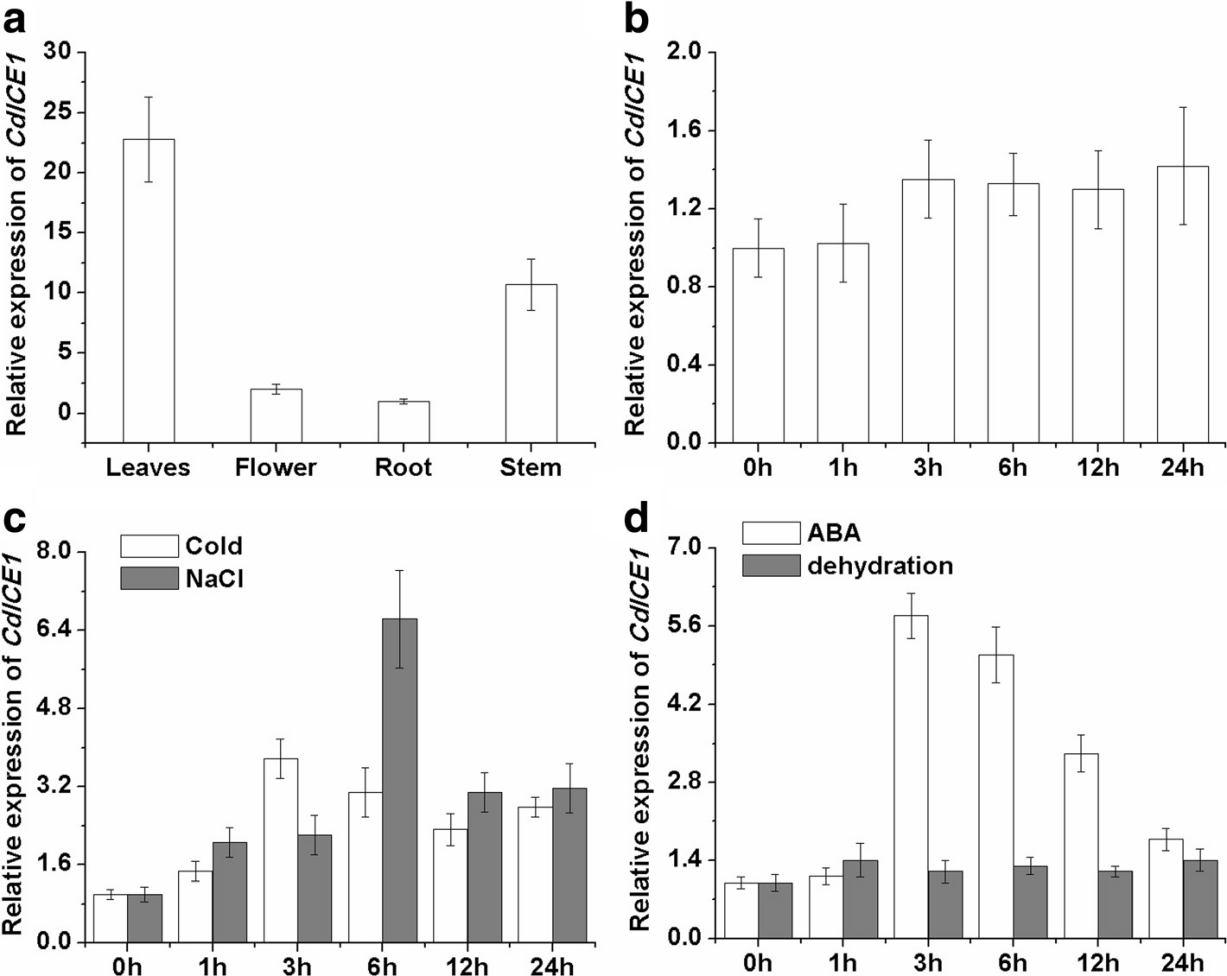
**Quantitative reverse transcription PCR (qRT-PCR) analysis of *CdICE1 *expression in *C. dichrum *plants. (a)***CdICE1* expression of different organs under non-stress conditions; **(b)** CdICE1 expression of leaves at different times under non-stress conditions; **(c–d)** CdICE1 expression of leaves under abiotic stress. Means and standard error calculated from triplicate assays.

A 1682-base pair promoter of *CdICE1* was isolated using the TAIL-polymerase chain reaction (PCR) method, described in Chen [43]. Its sequence including a few labeled stress-related *cis*-elements is shown in Additional file 1: Figure S1. Expression analysis of different tissues revealed its constitutive expression at different levels, with the strongest expression found in leaves and stems (Figure 1a). *CdICE1* was significantly upregulated by cold, NaCl and ABA but not by dehydration (Figure 1). Under cold and ABA stress, *CdICE1* expression increased gradually over the first three hours and then decreased slightly (Figure 1c, d). In addition, *CdICE1* expression was induced under salt stress, with expression peaking at six hours and then declining gradually (Figure 1c). No apparent inducible expression of *CdICE1* was detected with 20% polyethylene glycol (PEG) dehydration (Figure 1d).

### Subcellular localization of CdICE1:GFP fusion protein

**Figure 2:**
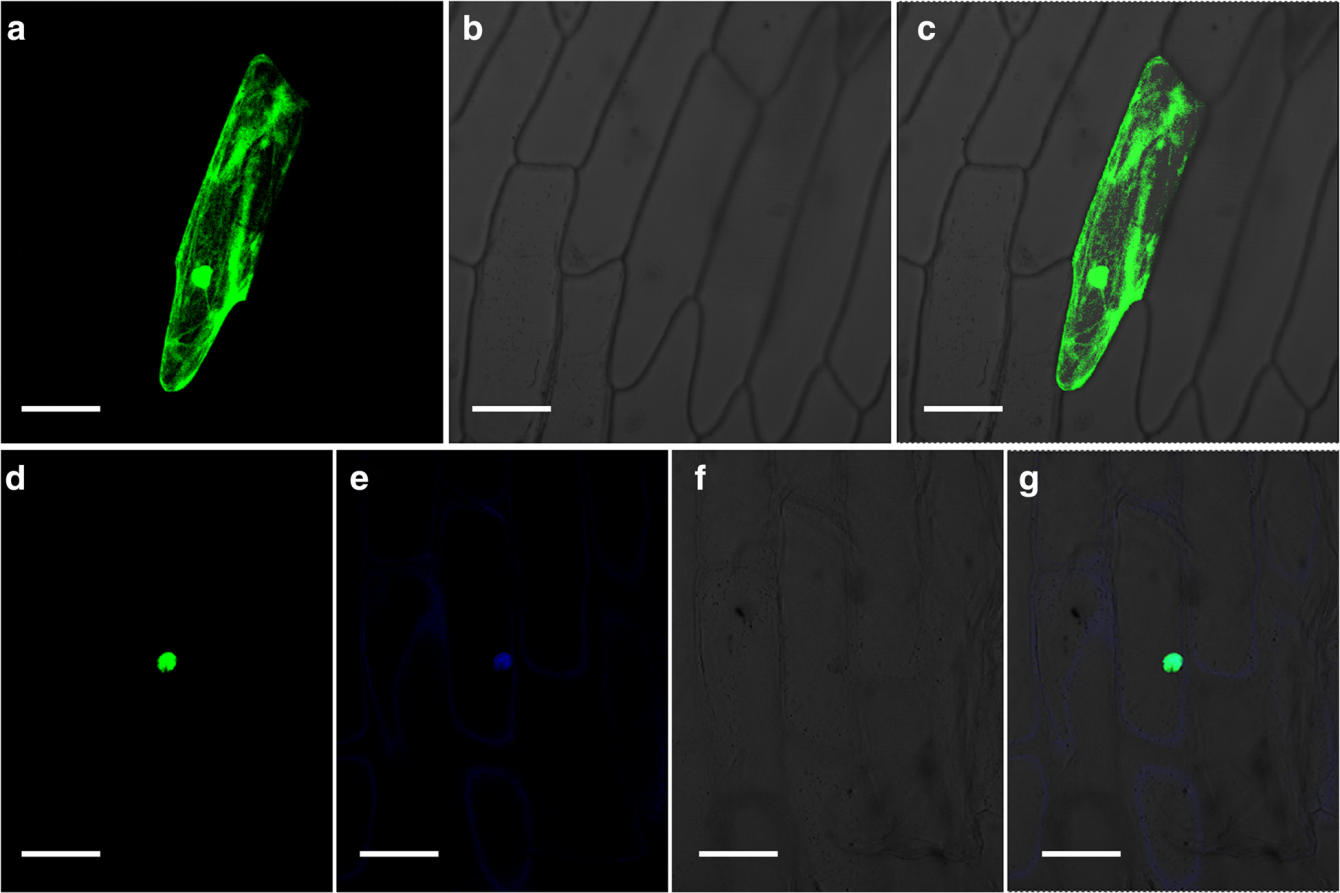
**Subcellular localization of CdICE1 protein in onion epidermal cells. (a–c)** Onion epidermal cells transformed with *35S::GFP* as a control; **(d–g)** Onion epidermal cells transformed with *35S::CdICE1-GFP*. Photographs were taken **(a, d)** under dark-field for green fluorescence; **e (b, f)** under bright-field for the morphology of the cells; (**e**, DAPI staining) under dark field for blue fluorescence; and **(c, g)** in combination. Bar = 50 μm. DAPI, 4',6-diamidino-2-phenylindole.

To explore the subcellular localization of CdICE1, we used a transient assay of CdICE1-GFP fusion constructs introduced into onion epidermal cells by particle bombardment. As shown in Figure 2, GFP alone resulted in diffused distribution of green fluorescence throughout the entire cell (Figure 2a-c). In contrast, CdICE1-GFP localized predominantly to the nucleus, which was further confirmed by 4',6-diamidino-2-phenylindole (DAPI) staining (Figure 2d-g).

### ICE1 binds to the MYC element in the CdDREBa promoter and activates transcription

**Figure 3:**
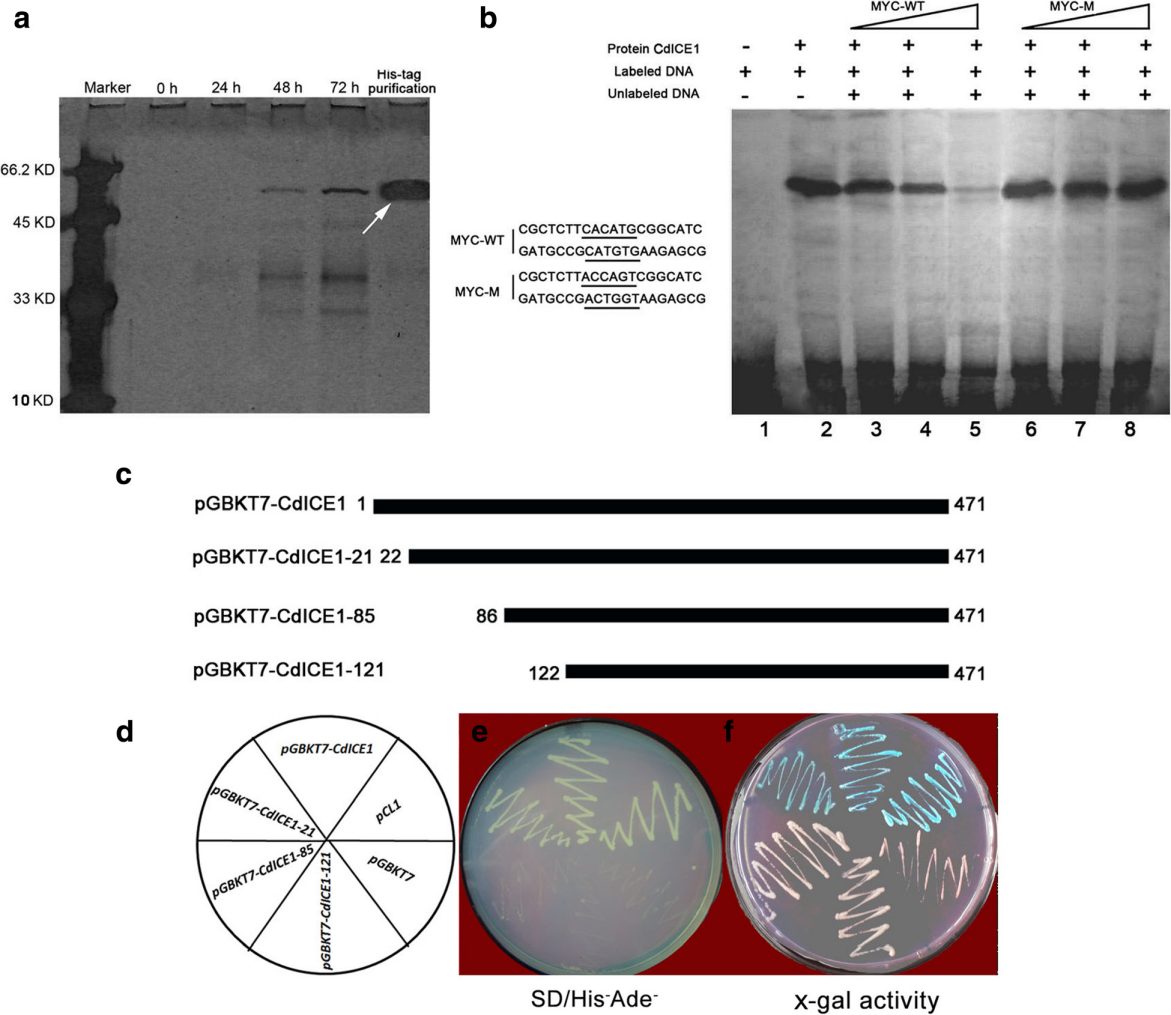
**Eukaryotic expression and EMSA assays of CdICE1 and transcriptional activation assays. (a)** Eukaryotic expression of CdICE1 and purification using His-tag; **(b)** Binding assays between CdICE1 protein and MYC elements: purified protein was incubated with 50 ng of biotin-labeled probe. Non-labeled probe with different concentrations (50, 100, and 500 times) was added; **(c)** Fragment constructs derived from different N’-deletions of amino acids; **(d)** Transcriptional activation assays of CdICE1 via yeast one-hybrid assays. EMSA, electrophoretic mobility shift assay.

To determine whether CdICE1 binds to MYC recognition sites in the *CdDREBa* promoter, CdICE1-His fusion protein was expressed and purified from *Pichia pastoris* (Figure 3a). MYC-WT sequences were used to determine the interaction with His–ICE1 in an electrophoretic mobility shift assay (EMSA). The results showed that the complex between CdICE1 and MYC-WT was inhibited by the MYC-WT competitor, but not by a mutated competitor (MYC-M) (Figure 3b), implying a specific interaction between CdICE1 and MYC elements.

A yeast one-hybrid system was used to investigate CdICE1 transcriptional activity. Yeast cells harboring pCL1, pGBKT7-CdICE1 or pGBKT7-CdICE1-21 proliferated on SD/His^-^Ade^-^ medium and X-α-gal activity was detected based on blue coloration, unlike pGBKT7, pGBKT7-CdICE1-85 or pGBKT7-CdICE1-121 (Figure 3c-f). The data confirmed that CdICE1 was associated with transactivation activity, suggesting that N’ 22–85 amino acids may be required for transactivation.

### **CdICE1 positively regulates CBFs associated with freezing tolerance in *Arabidopsis *** **at 4°C**

**Figure 4:**
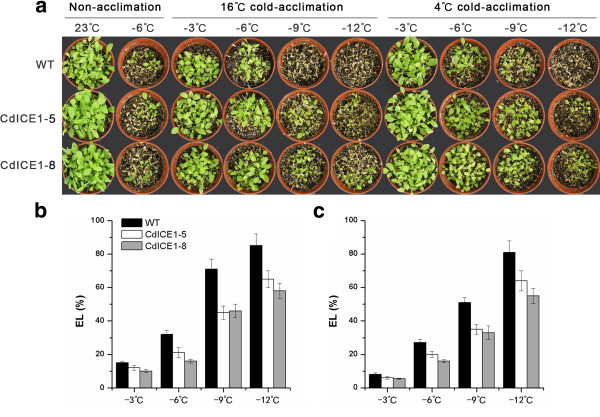
**Overexpression of *CdICE1 *in *Arabidopsis *improves freezing tolerance. (a)** Freeze-treated *Arabidopsis* after 21-day recovery growth; **(b)** Electrolyte leakage of *Arabidopsis* leaves under different freezing treatments after acclimation at 16°C; **(c)** Electrolyte leakage of *Arabidopsis* leaves under different freezing treatments after acclimation at 4°C.

Transgene overexpression was detected using genomic DNA PCR and mRNA quantitative reverse transcription PCR (qRT-PCR) assays (see Additional file 2: Figure S2) in transgenic plants but not wild-type (WT) *Arabidopsis*. Independent T_3_ homozygous transformants CdICE1-5 and CdICE1-8 were used for further experiments in freezing tolerance. After freezing treatments (−6°C), no significant differences in electrolyte leakage (EL) or survival rate were observed between transgenic and non-transgenic plants that were not initially subjected to acclimation (Figure 4). In both transgenic and WT plants, 4°C or 16°C temperature acclimation enhanced freezing tolerance, but plants overexpressing *CdICE1* showed higher survival rates after recovery and lower EL than WT ones under both freezing treatments (Figure 4). These data indicate that *CdICE1* plays an important role in cold acclimation-mediated freezing tolerance.

**Figure 5:**
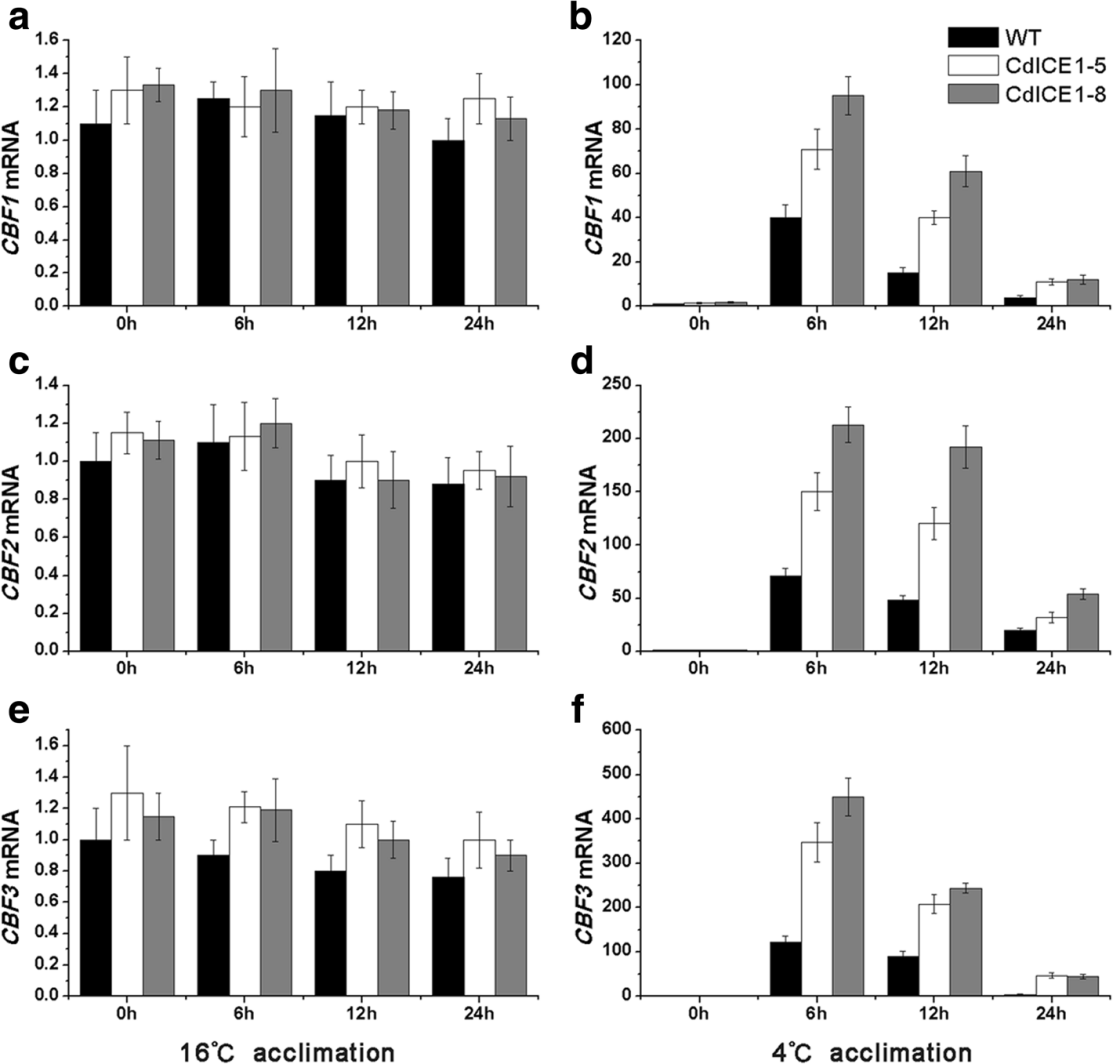
**Quantitative reverse transcription PCR (qRT-PCR) analysis of CBFs between WT and transgenic *Arabidopsis *plants. (a, c, e)**: CBF1-3 mRNA expression levels during acclimation at 16°C; **(b, d, f)**: CBF1-3 mRNA expression levels during acclimation at 4°C. WT, wild type.

**Figure 6:**
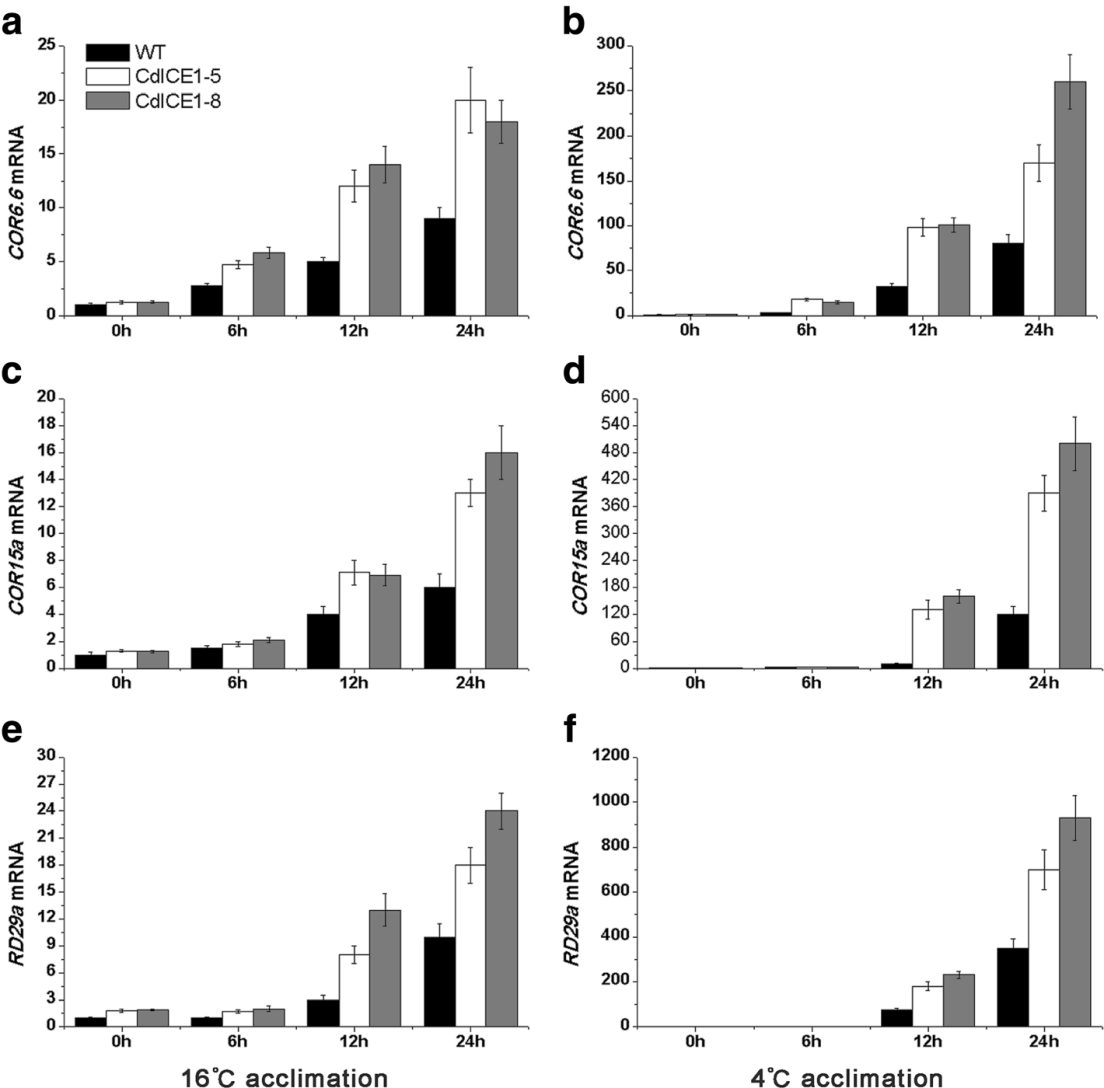
**Quantitative reverse transcription PCR (qRT-PCR) analysis of downstream freezing-tolerance genes *CORs *and *RD29a *between WT and transgenic *Arabidopsis *plants. (a, c, e)**: mRNA expression levels at 16°C; **(b, d, f)**: mRNA expression levels under conditions below 4°C. WT, wild type.

Downstream genes of *CdICE1* were further tested using qRT-PCR to determine changes in freezing tolerance in transgenic *Arabidopsis*. Acclimation at 16°C did not affect expression of the three *CBF* genes compared with their levels at 23°C. *CdICE1* overexpression did not alter the transcription of CBF1 or 2 but there was a slight increase in CBF3 (Figure 5a,c,e). Acclimation at 4°C induced their expression, with transgenic plants showing higher *CBF* expression than WT plants (Figure 5b,d,f). In two transgenic plants, *CBF3* expression was higher compared with the other two genes (Figure 5d,f). Expression of the three *COR* genes was also significantly higher in *CdICE1*-overexpressing lines compared with WT plants undergoing acclimation at 16°C or 4°C (Figure 6). These data indicate that during acclimation at 4°C, *CdICE1* mediates freezing tolerance via a CBF-COR pathway, consistent with a previous report [1]. Acclimation at 16°C was associated with an improved mechanism of *CdICE1* to freezing tolerance inconsistent with acclimation at 4°C.

### CdICE1 negatively regulates the miR398-CSD pathway involved in freezing tolerance in *Arabidopsis* under acclimation at 16°C

**Figure 7:**
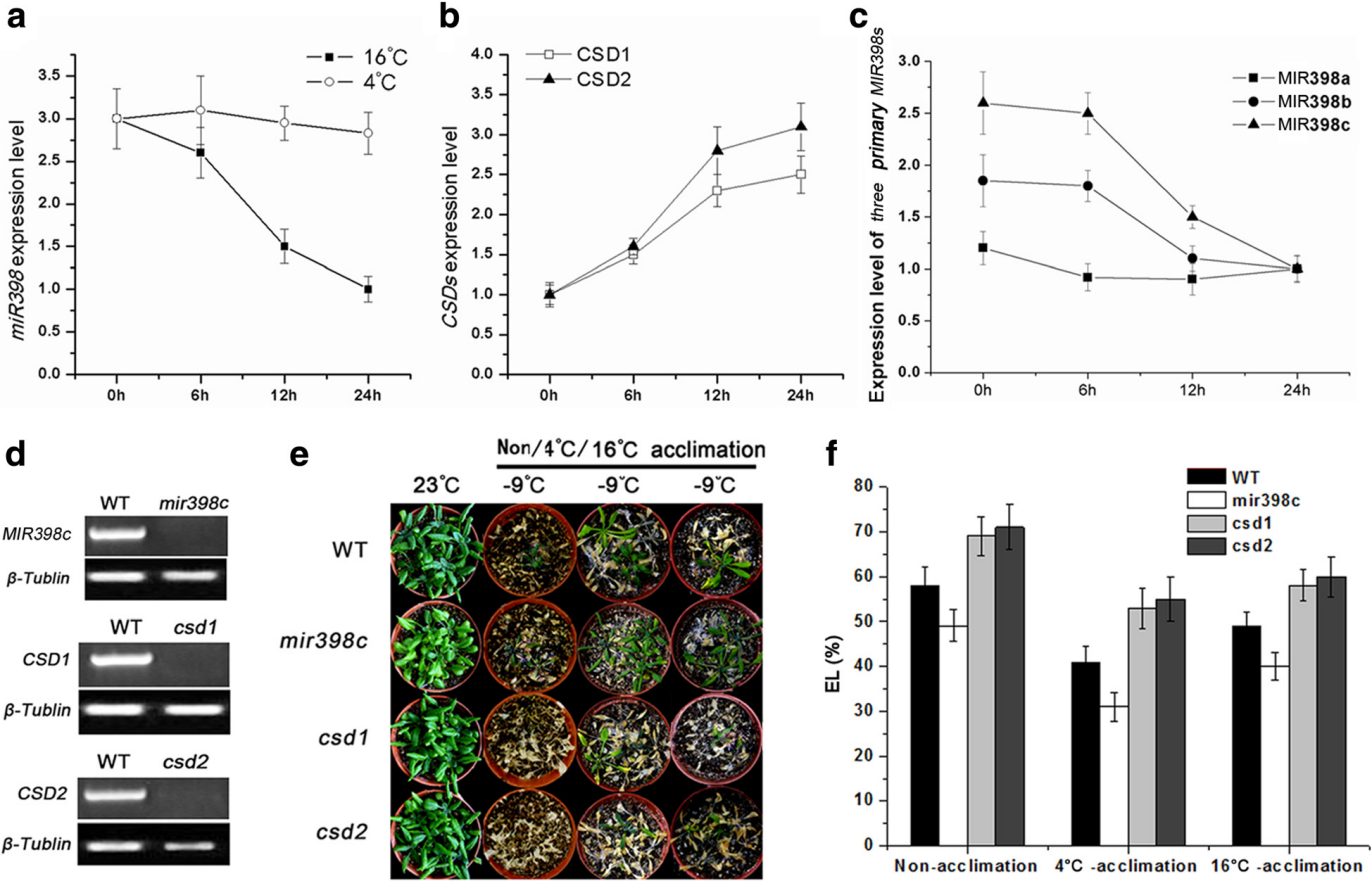
**Quantitative reverse transcription PCR (qRT-PCR) analysis and comparison of freezing tolerance between WT and mutant *Arabidopsis*. (a)** matured *miR398* expression under acclimation at 16°C and 4°C; **(b)***CSD1* and *CSD2* expression at 16°C; **(c)** qRT-PCR analysis of primary transcript *MIR398 a*, *b* and *c* during acclimation at 16°C; **(d)** RT-PCR analysis of expression of *MIR398c*, *CSD1* and *CSD2* between WT *Arabidopsis* and the mutants; **(e)***Arabidopsis* treated at −9°C after 21 days of recovery growth following 16°C/4°C/non-acclimation conditions; **(f)** Electrolyte leakage (EL) of *Arabidopsis* leaves at 1 hour after treatment at −9°C. WT, wild type.

To further explore the regulatory mechanism of acclimation to 16°C that induces freezing tolerance in transgenic *Arabidopsis*, miR398 and CSD expression levels were studied. In WT plants, *miR398* expression was downregulated twofold during acclimation at 16°C for 24 hours, while no changes occurred under 4°C (Figure 7a). The downregulation due to silencing of all three MIR398 loci was further examined. RT-PCR showed that primary *MIR398b (c)* exhibited significant decreases and no alteration in primary *MIR398a* expression at 16°C (Figure 7c). The two target genes *CSD1* and *CSD2* were upregulated 1.5- and 2.1-fold, respectively, after 24 hours at 16°C (Figure 7b).

To elucidate the association of miR398-CSDs with freezing tolerance, we examined phenotypes of growth recovery after freezing treatment in the three *Arabidopsis* mutants *mir398c*, *csd1*, and *csd2* (Figure 7d-f). After freezing treatment, *mir398c* mutants exhibited higher survival rates, whereas *csd1* and *csd2* showed lower survival compared with WT plants. Freezing tolerance of *mir398c*, *csd1* and *csd2* was induced at 4°C/16°C compared with the non-acclimatized plants (Figure 7e). The EL values in *mir398c* were always lower while those in *csd1* and *csd2* were higher than the WT plants (Figure 7f).

**Figure 8:**
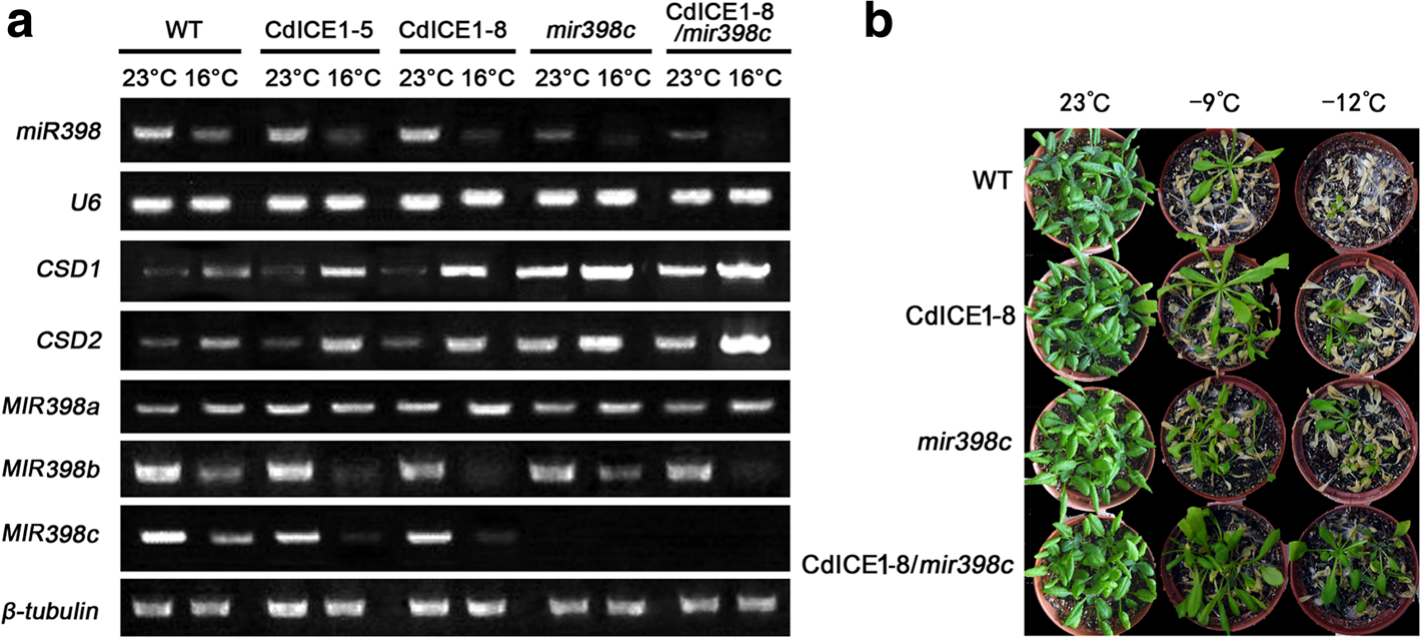
**Expression and freezing tolerance analysis. (a)** RT-PCR analysis of *miR398*, *MIR398a*, *b*, *c*, *CSD1*, and *CSD2* of WT, *CdICE1* transgenic *Arabidopsis* and crossing materials *CdICE1-8/ mir398c*; **(b)** freezing tolerance of WT, *mir398c* mutant and *CdICE1-8/mir398c* after acclimation at 16°C. WT, wild type.

Expression levels of *CSD1* and *CSD2* were much higher in *mir398c* mutants compared with WT plants (Figure 8a). Under non-acclimatized conditions, *CdICE1*-overexpressing plants were no different than WT with respect to expression of *miR398* and its two target genes *CSD1* and *CSD2* (Figure 8a). After 24 hours of acclimation at 16°C, however, *miR398*, *MIR398b* and *MIR398c* in transgenic plants was downregulated compared with WT, and *CSD1* and *CSD2* were upregulated (Figure 8a). The freezing tolerance of CdICE1-8/*mir398c* plants (a cross between *CdICE1*-overexpressing plants and *mir398c*) was higher than *mir398c* after acclimation at 16°C (Figure 8b), which may partially result from primary *MIR398b* reduction by CdICE1.

## Discussion

The *CdICE1* gene isolated from *C. dichrum* encodes a bHLH protein with an amino acid sequence highly similar to that of *Arabidopsis* ICE1 and ICE2. Its over-expression increases tolerance to low temperature, drought and salinity stress in chrysanthemum [42]. However, the molecular mechanism underlying freezing tolerance is not well understood. In this study, *CdICE1* expression was induced by cold, salt and ABA, and its nuclear location was confirmed by subcellular localization *in vivo*. BHLH proteins regulate downstream genes through sequence-specific interactions in the promoter regions [2,44]. In *Arabidopsis* and wheat, ICE1 acts directly upstream of *CBFs* by binding to the MYC recognition sites present in CBF gene promoters, and then subsequently triggers expression of *CBF/DREB* regulons [1,45]. Based on EMSA in the current study, the CdICE1 protein was found to bind to the MYC recognition site of the *CdDREBa* promoter. Overexpression of *CdDREBa* has been previously observed to improve drought and salinity stress tolerance in chrysanthemums [46]. In our study, CdICE1 protein also showed transactivation activity. These results indicate that an ICE1-DREB pathway exists in *C. dichrum* and that *CdICE1* might be a useful transcription factor in chrysanthemum breeding for improvement of stress tolerance.

The CBF cold-responsive pathway is an important cold-acclimation gene network that contributes to cold tolerance [15,17,19,47]. A dominant mutation in ICE1 blocks cold induction of the *CBF3* regulon and impairs freezing tolerance [1]. In contrast, ICE1 overexpression increases cold tolerance relative to WT plants not only in *Arabidopsis* but also in rice and apples [1,2,23,48]. In this study, *CdICE1* overexpression resulted in elevated expression of *CBF* and three *COR* genes during 4°C cold acclimation and enhanced tolerance to freezing stress. These data suggest that CdICE1 acts as a signal transduction component in the CBF pathway and is associated with cold tolerance, similar to *ICE* genes in *Arabidopsis* and wheat [1,45].

After the discovery of miRNAs, researchers recognized the important role of these small RNAs in abiotic stress response via posttranscriptional gene regulation [35,49]. In Chinese cabbage (*Brassica rapa*), extremely high temperature (46°C) reduces the *miR398* level [40]. The expression of *miR398* was also steadily decreased over 48 hours under salt stress in *Arabidopsis*[38]. Our study found that the downregulation of miR398 is involved in changes associated with a 23°C to 16°C temperature shift (Figure 7c), which is consistent with a previous report suggesting that the expression of *miR398* was significantly lower under ambient temperature (16°C) than at 23°C in *Arabidopsis*[39]. It has been shown that expression of *MYB*, *WRKY* and *bHLH* family genes is regulated by ICE1 in *Arabidopsis*[48]. Cis-elements such as MYB, WRKY or bHLH transcription factor-binding elements were predicted in MIR398b and MIR398c promoters (see Additional file 3: Figure S3). Therefore, we propose that CdICE1 decreased the miR398 level via ICE1-dependent Transcription factors (such as MYB, WRKY or bHLH) that act as repressors of pri-miR398b (c).

In *Arabidopsis*, miR398-CSDs were found to participate in regulation of biotic stress (due to *Pseudomonas syringae*) and abiotic stresses such as Cu^2+^, UV, ozone, salt, ABA and heat [35,37,38,41]. Transgenic *Arabidopsis* plants overexpressing a miR398-resistant form of *CSD2* accumulate more *CSD2* mRNA than plants overexpressing a regular *CSD2.* The transgenic plants were more tolerant to increased radiation, heavy metals and other oxidative stresses [35]. In this study, the downregulated expression of *miR398* at 16°C induced expression of two *CSD* genes (Figures 7c and 8a). Freezing tolerance was negatively regulated by *miR398* levels and improved by *CSD* genes, consistent with a previous study demonstrating that *CSD* overexpression elevated the freezing tolerance of alfalfa [50]. *CdICE1-*overexpressing plants induced higher ROS content compared with WT plants during the initial stages (6 hours and 12 hours) of acclimation to 16°C, while no significant difference in ROS levels was observed between WT and transgenic plants after 24 hours at 16°C (see Additional file 4: Figure S4). We speculate that early induction of ROS might act as a signal to reduce *miR398* expression in response to acclimation at 16°C. The reduction in miR398 in turn increased the expression of *CDS1* and *CSD2* that eliminated the ROS with similar ROS levels between WT plants and *CdICE1*-overexpressing plants after 24 hours at 16°C (see Additional file 4: Figure S4). In addition, freezing tolerance assays of WT, mir398c mutant and CdICE1/mir398c (a cross of *CdICE1* overexpressing plants with mir398c mutant) at 16°C showed that CdICE1/mir398c plants were more tolerant compared with the mir398c mutant (Figure 8b), suggesting that down regulation of MIR398c was one of the ICE1-regulated pathways. Taken together, overexpression of *CdICE1* resulted in a decrease in *miR398* expression levels following transition from 23°C to 16°C, indicating that CdICE1 induced freezing tolerance partially via the miR398-CSD pathway.

## Conclusions

The different alterations in ambient temperature resulted in improvement in freezing tolerance. Upon transition from 23°C to 4°C, the ICE family genes played an important role in inducing the expression of CBF genes, consistent with past results. However, interesting data showed that *CdICE1* from *C. dichrum* regulated freezing tolerance of *Arabidopsis* partly through the miR398-CSD pathway following transition from 23°C to 16°C.

## Methods

### Plant materials and treatment

*C. dichrum* plants were obtained from the Chrysanthemum Germplasm Resource Preserving Centre, Nanjing Agricultural University, China. We subjected three-week-old seedlings of *C. dichrum* to varying durations of abiotic stress treatments, including 200 mM NaCl, 20% PEG, 100 μM ABA and 4°C temperature, to analyze the expression pattern.

A *pEarleyGate103-CdICE1* expression plasmid was introduced into *Agrobacterium tumefaciens* strain EHA105 and used for transgenic measurements in *Arabidopsis* ecotype Columbia using the floral dip method [51,52]. *Arabidopsis* mutants *mir398c* (SALK_038698C), *csd1* (SALK_024857C), and *csd2* (SALK_041901C) were obtained from the Arabidopsis Biological Resource Center (Columbus, OH, USA). We obtained the *CdICE1-*overexpressing *Arabidopsis* T_3_ plants (CdICE1-5 and CdICE1-8) and CdICE1-8/*mir398c* plants by crossing CdICE1-8 T_3_ plants with *mir398c* (SALK_038698C). *Arabidopsis* plants were grown in soil at 23°C and 70% relative humidity under 24-hour constant light (100 μmol m^-2^ sec^-1^) for 15 days. The plants were then shifted to either 4°C or 16°C for 24 hours followed by freezing at -3, -6, -9 or -12°C for 1 hour. After freezing treatment, plants were incubated at 4°C for 1 day and then returned to 23°C. EL from leaves was assayed and survival rate was determined 21 days later.

### Subcellular localization of CdICE1 proteins

The full-length coding region of *CdICE1* was fused to the N-terminus of a green fluorescent protein (GFP) gene under the control of a CaMV 35S promoter. The *CdICE1* open reading frame (ORF) fragment harboring *Bam*HI and *Sma*I enzyme sites was amplified with the primer pair CdICE1-B/CdICE1-S (Table 1). Following purification, the resulting PCR product was cut by *Bam*HI and *Sma*I and ligated to the same cleavage site on pBI121-GFP using T4 DNA ligase. Plasmid DNA pBI121-CdICE1-GFP was transiently introduced into onion epidermal cells using a helium-driven particle accelerator (PDS-1000; Bio-Rad; Hercules; California; USA). An empty vector with GFP (pBI121-GFP) was transformed into another set of epidermal cells as a control. After bombardment, onion peels were kept on MS plates in the dark for 16 hours. Confocal laser microscopy (Leica SP2) was used to monitor GFP expression.

**Table 1:** Primer sequences

**Gene**	**Oligo names**	**Primer sequences 5′-3′**
	CdICE1-B	ACTGGATCCATGCTACCGGAAAACGACA
	CdICE1-S	TCCCCCGGGAATGGCACCATGATAACCT
	zh-F	CGGGTACCATGCTACCGGAAAACGACA
	zh-R	TCCCCGCGGTAATGGCACCATGATAACCT
	ICE1	ACTCATATGATGCTACCGGAAAACGACA
	ICE21	ACTCATATGACCCCTACAACCACCCCA
	ICE85	ACTCATATGTCACCTTCACAATCACACTCAC
	ICE121	ACTCATATGTGTGACCCTAGCTTCATTTCCA
	ICE471	ACTGGATCCAATGGCACCATGATAACCT
*CdICE1*	CdICE-F	CCTACCCAACAAATGGCAACTA
	CdICE-R	TCTCTCCACTCTGTCTCAGCGC
*CBF1*	cbf1-F	GTGACGTGTCGCTTTGGAGTTAC
	cbf1-R	GTGAAGCAAAGAAGTAGAAAACG
*CBF2*	cbf2-F	TCGAGGGAGATGATGACGTGTCC
	cbf2-R	TATTTTGATTTGTTGCTTATGG
*CBF3*	cbf3-F	CGACGGCGATGATGACGACGT
	cbf3-R	GCATTTAAGAATAGCCCACAC
*COR6.6*	cor6.6-F	CAGAGACCAACAAGAATGCC
	cor6.6-R	CGATATACTCTTTCCCGCCT
*COR15a*	cor15a-F	AAAGCAGGAGAGGCTAAGGAT
	cor15a-R	CATGAAGAGAGAGGATATGGATCA
*RD29a*	rd29a-F	TAGGAAAGTAAAGGCTAGAGCTAAG
	rd29a-R	AATCGGAAGACACGACAGG
*β-tubulin*	tubin-F	AAGATTCGTCCCACGCG
	tubin-R	TCCTTTAGCCCAATTGTTACC
*U6*	U6-F	CTCGCTTCGGCAGCACA
	U6-R	AACGCTTCACGAATTTGCGT
*CSD2*	CSD2-F	AACCCTAACAACATGACACACG
	CSD2-R	GAACCACAAAGGCTCTTCCAAC
*CSD1*	CSD1-F	AGACCCTGATGACCTCGGAAA
	CSD1-R	GCCACACACCAGAAGATACAC
*miR398*	miR398-F	TGTGTTCTCAGGTCACCCCT
	Uni-miR qPCR Primer	From TaKaRa
MIR398a	miR398a-F	AGAAGAAGAGAAGAACAACAGGAGGTG
	miR398a-R	TTTAGTAAGGTGAAAAAATGGAACAGG
MIR398b	miR398b-F	TAACAAGAAGATATCAATATATCATG
	miR398b-R	ACCATTTGGTAAATGAGTAAAAGCCAGCC
MIR398c	miR398c-F	TCGAAACTCAAACTGTAACAGTCC
	miR398c-R	ATTTGGTAAATGAATAGAAGCCACG

### Electrophoretic mobility shift assay

Primers zh-F and zh-R (Table 1), containing *Kpn*I and *Sac*II restriction sites, were used to amplify the CdICE1 ORF. After digestion with *Kpn*I and *Sac*II, the amplified fragment was inserted into the expression vector *pPICZαA* (Invitrogen, Carlsbad, California, USA). The resulting expression vector was transformed into the yeast strain X33 following digestion with *Sac*I. The CdICE1 protein was prepared according to the manufacturer’s instructions. EMSA was carried out using a LightShift Chemiluminescent EMSA kit (HyClone-PIERCE, Rockford, Illinois, USA). The double-stranded oligonucleotides MYC-WT (CGCTCTTCACATGCGGCATC) from the *CdDREBa* promoter and mutated MYC-M (CGCTCTTACCAGTCGGCATC) were used as probes and competitors for the EMSA.

### Transactivation activity analysis of CdICE1

Four different N’-deletion variants of the CdICE1 coding region were amplified using forward primers ICE1, ICE21, ICE85 or ICE121 and reverse primer ICE471 (Table 1). The resulting fragments were inserted into the *Nde*I/*Bam*HI cloning site of the yeast expression vector pGBKT7 to produce pGBKT7-CdICE1, pGBKT7-CdICE1-21, pGBKT7-CdICE1-85 and pGBKT7-CdICE1-121 (Figure 4). Each of these constructs, pCL1 (positive control) and pGBKT7 (negative control) were individually introduced into separate cultures of *Saccharomyces cereviseae* strain Y2H Gold (Clontech, Mountain View, California, USA) following the manufacturer’s protocol. Selection of transformants carrying either pGBKT7-CdICE1/-21/-85/-121 or pGBKT7 was carried out on SD/-Trp medium. The pCL1 transformants were selected on SD/Leu^-^ medium. All six transformant cell lines were then transferred to an SD/His^-^Ade^-^ and SD/X-α-gal medium to observe cell growth.

### Gene expression analysis using quantitative real-time PCR (qRT-PCR) and RT-PCR analysis

Total RNA was extracted from *C. dichrum* and *Arabidopsis* using a plant RNAiso plus kit (Takara, Otsu, Japan), followed by RNase-free DNase I treatment to remove genomic DNA. First-strand cDNA synthesis from 1 μg total RNA of *C. dichrum* was accomplished by reverse transcription using SuperScript III reverse transcriptase (Invitrogen). *Arabidopsis* RNA was synthesized to cDNA with a One Step PrimeScript miRNA cDNA synthesis kit (Takara). To determine the transcription levels of different genes, a SYBR Green PCR kit (TOYOBO, Osaka, Japan) was employed along with the gene specific primers listed in Table 1. Transcription levels of either *EF1α* (*C. dichrum* translation elongation factor gene) or *β-tubulin* (*Arabidopsis*) were used as internal references. Matured miR398 expression levels were assayed with the specific primers miR398-F/Uni-miR qPCR primer compared with the reference gene *U6* (primer U6-F/-R). PCRs were conducted according to the following protocol: 60 seconds denaturation at 95°C, 30 seconds annealing at 55°C and 30 seconds elongation at 72°C for 40 cycles. RT-PCR conditions for primary *MIR398b* and *MIR398c* transcript amplification followed the method described by Sunkar [35]. Briefly, the PCR conditions included: 95°C for 15 minutes, 30 seconds denaturation at 94°C, 30 seconds annealing at 60°C and 2 minutes elongation at 72°C for 35 cycles, 72°C for 10 minutes. RT-PCR conditions for *MIR398a, CSD1* and *CSD2* amplification were similar to *MIR398b* and *MIR398c* except that PCR cycles were 50, 30 and 30, respectively. Three replicates of each reaction were performed. The data were analyzed by Bio-Rad iQ5 Optical System Software v1.0.

### Determination of O_2_^–^ production and H_2_O_2_ content

Superoxide anion production was measured following the method of Frahry [53] and the content of H_2_O_2_ was determined, according to Bellincampi [54].

## Competing interests

The authors declare that they have no competing interests.

## Authors’ contributions

YC, JJ and FC conceived the study and designed the experiments. YC, AS, SC, HS and LZ performed the experiments. YC, HL, CG and JS analyzed the data with suggestions by JJ, FC and WF. YC and JJ wrote the manuscript. All authors read and approved the final manuscript.

## Supplementary Material

Additional file 1: Figure S1 Analysis of the promoter sequence of *CdICE1* and responsive elements. Note: Functional elements as predicted by PLACE software (http://www.dna.affrc.go.jp/PLACE/signalscan.html) are either underlined or shaded. Click here for file

Additional file 2: Figure S2 PCR identification of resistant T1 generation plants. **(a)** PCR assays for *CdICE1* at the genome level; **(b)** QRT-PCR assays of *CdICE1* expression in transgenic plants. Click here for file

Additional file 3: Figure S3 Prediction of the promoter sequence of MIR398b and MIR398c and responsive elements. Note: Functional elements as predicted by PLACE software (http://www.dna.affrc.go.jp/PLACE/signalscan.html) are underlined. Click here for file

Additional file 4: Figure S4 ROS assays in WT and *CdICE1* overexpressing plants under 16°C. **(a)** O_2_^–^ content; **(b)** H_2_O_2_ content. Asterisk indicates significant difference at *P* <0.05 compared with the WT plants by Duncan’s test. Click here for file
