# Supplementary figures and images for: Ambient temperature enhanced freezing tolerance of Chrysanthemum dichrum CdICE1 Arabidopsis via miR398
Source: BMC Biol. 2013 Dec 19;11:121. doi: 10.1186/1741-7007-11-121 (PMC3895800; doi:10.1186/1741-7007-11-121)

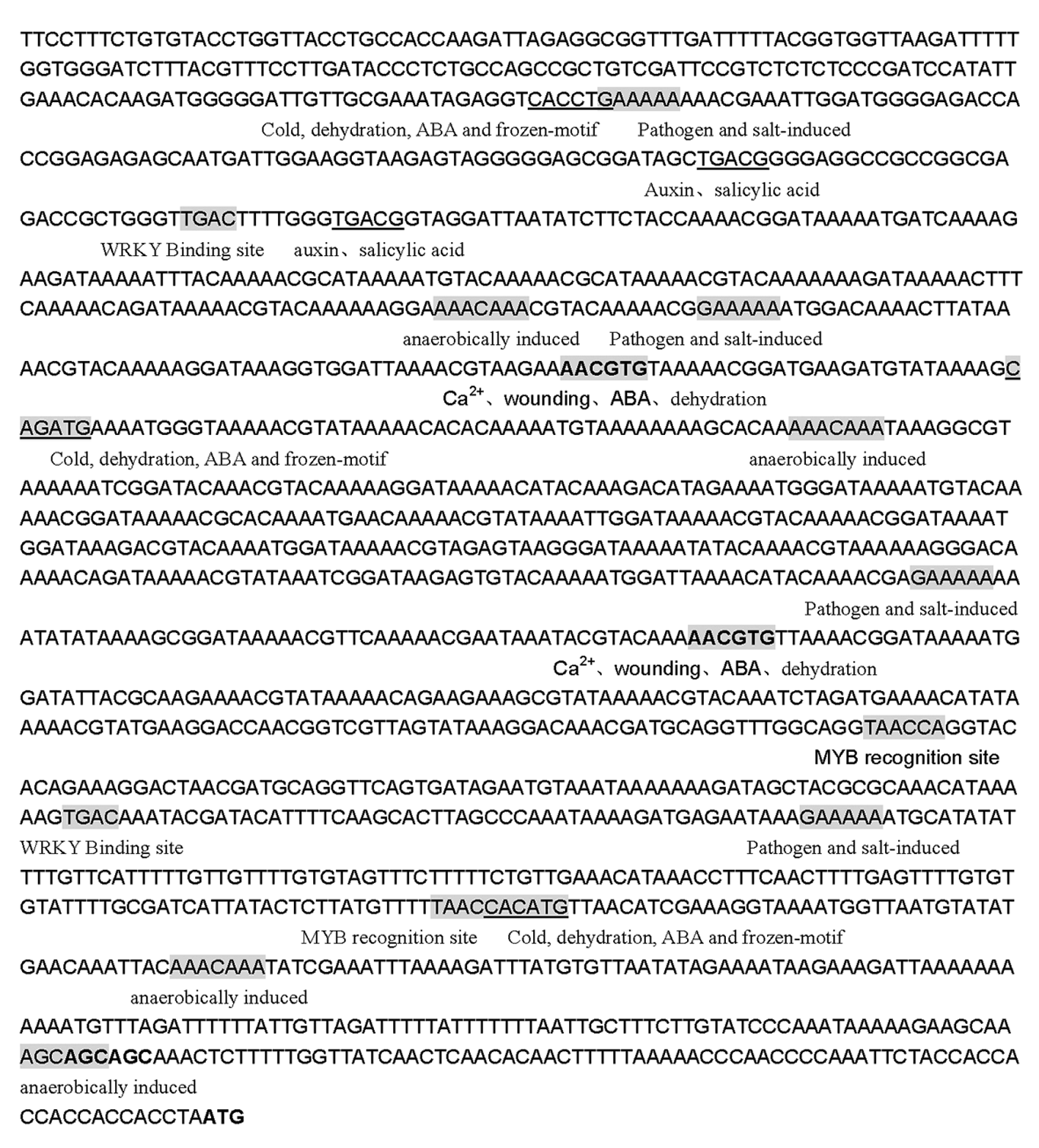

Supplement: Additional file 1: Figure S1 — Analysis of the promoter sequence of CdICE1 and responsive elements. Note: Functional elements as predicted by PLACE software (http://www.dna.affrc.go.jp/PLACE/signalscan.html) are either underlined or shaded. [file 1741-7007-11-121-S1.tiff]

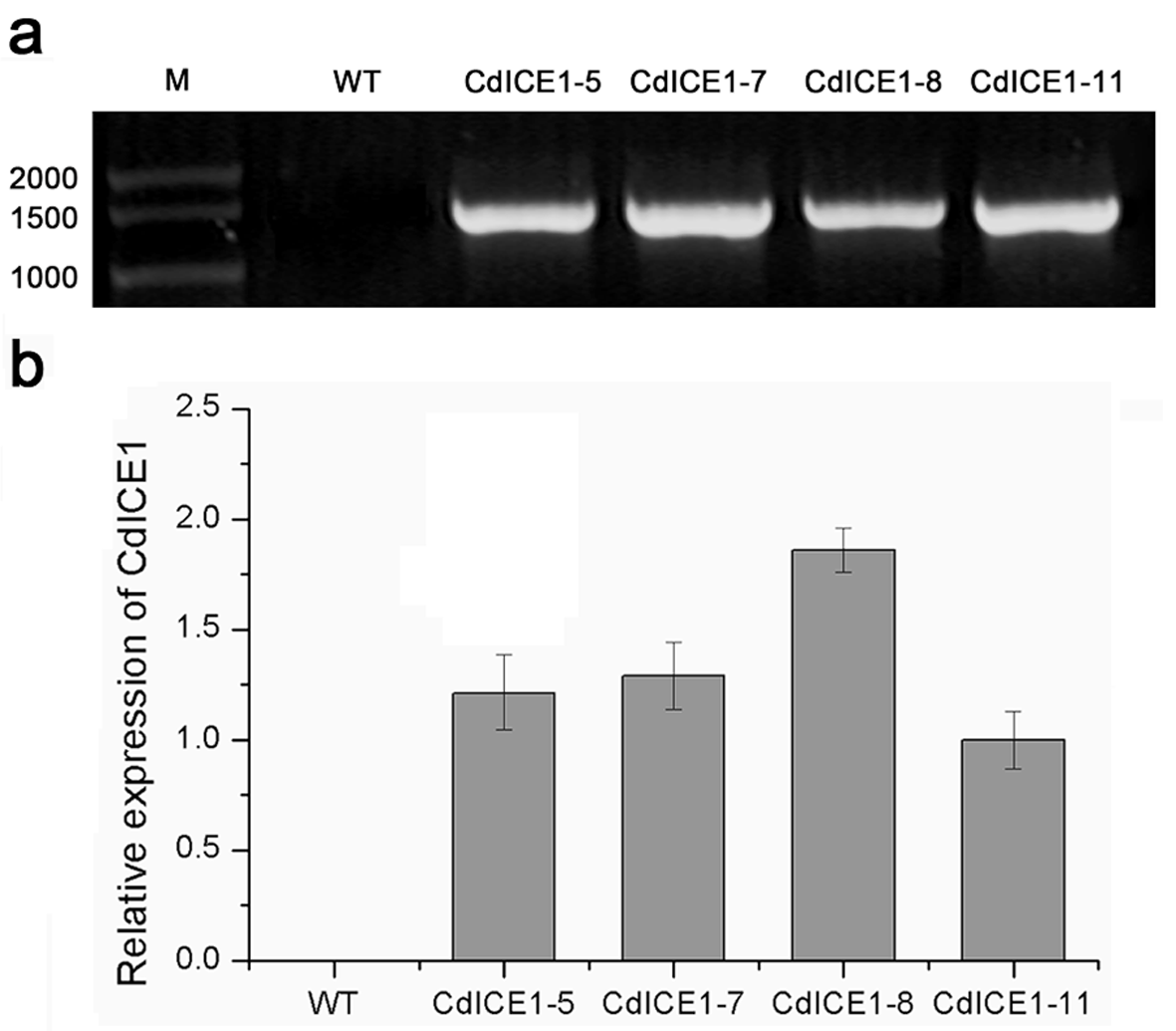

Supplement: Additional file 2: Figure S2 — PCR identification of resistant T1 generation plants. (a) PCR assays for CdICE1 at the genome level; (b) QRT-PCR assays of CdICE1 expression in transgenic plants. [file 1741-7007-11-121-S2.tiff]

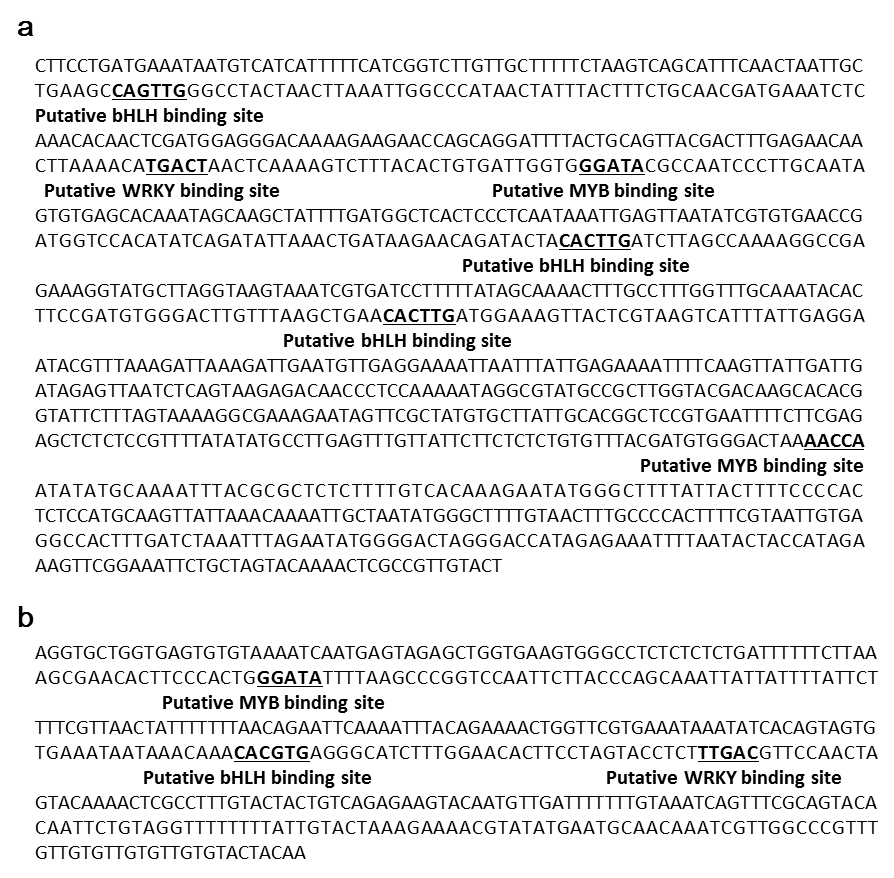

Supplement: Additional file 3: Figure S3 — Prediction of the promoter sequence of MIR398b and MIR398c and responsive elements. Note: Functional elements as predicted by PLACE software (http://www.dna.affrc.go.jp/PLACE/signalscan.html) are underlined. [file 1741-7007-11-121-S3.tiff]

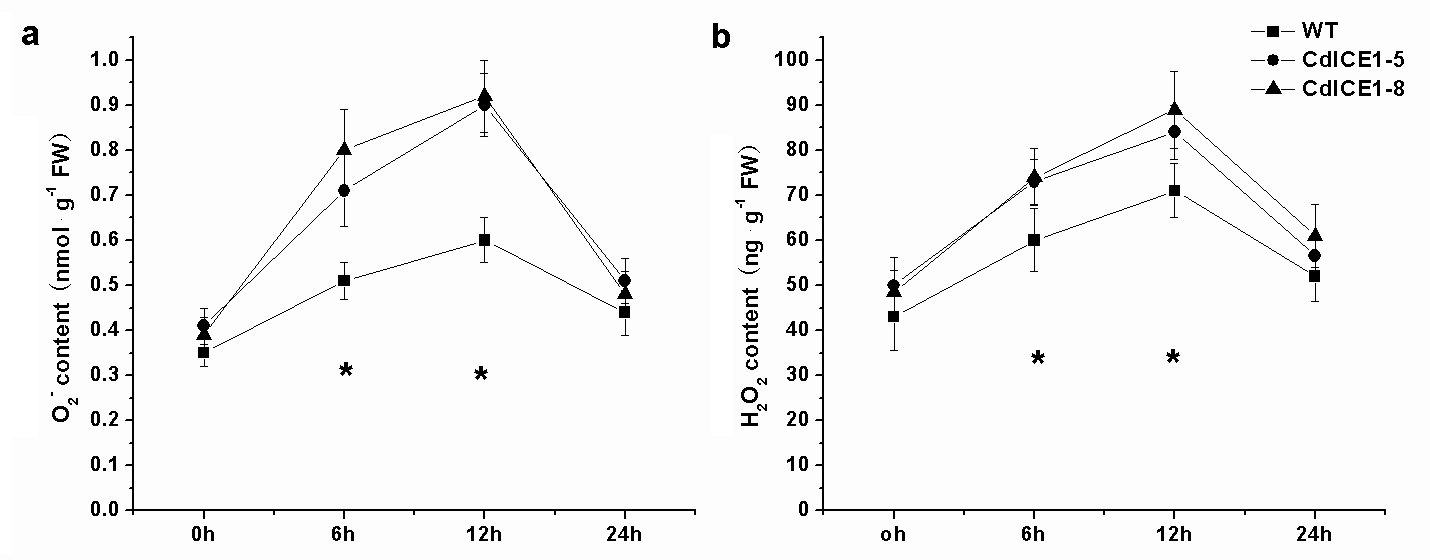

Supplement: Additional file 4: Figure S4 — ROS assays in WT and CdICE1 overexpressing plants under 16°C. (a) O2– content; (b) H2O2 content. Asterisk indicates significant difference at P <0.05 compared with the WT plants by Duncan’s test. [file 1741-7007-11-121-S4.tiff]
